# Health promotion and disease prevention: Advanced Practice Nursing competencies in women’s health consultations

**DOI:** 10.1590/0034-7167-2025-0064

**Published:** 2026-07-27

**Authors:** Jaqueline Martins Ramos, Daiana Bonfim, Andrea Liliana Vesga-Varela, Manoel Vieira de Miranda

**Affiliations:** I Hospital Israelita Albert Einstein, Faculdade Israelita de Ciências da Saúde Albert Einstein. São Paulo, São Paulo, Brazil; II Hospital Israelita Albert Einstein. São Paulo, São Paulo, Brazil; III Universidade de São Paulo. São Paulo, São Paulo, Brazil

**Keywords:** Advanced Practice Nursing, Primary Care Nursing, Primary Health Care, Health Promotion, Women’s Health., Enfermería de Práctica Avanzada, Enfermería de Atención Primaria, Atención Primaria de Salud, Promoción de la Salud, Salud de la Mujer.

## Abstract

**Objectives:**

to analyze Advanced Practice Nursing competencies in the domain of health promotion and disease prevention during women’s health nursing consultations in Primary Health Care.

**Methods:**

multicenter, descriptive, exploratory, cross-sectional study with a quantitative and qualitative approach, conducted in four Brazilian municipalities in the Northern, Northeastern, and Southeastern regions. In 2022 (May-July), 22 nurses conducted 69 recorded consultations. Data were organized in Excel and analyzed using descriptive statistics and content analysis.

**Results:**

contraceptive methods were offered (30.3% of consultations); counseling on sexually transmitted infections (2.86%); health promotion strategies (49.25%), without printed educational materials; disease screening according to protocols (47.16%); no tertiary prevention actions were identified; lifestyle changes were encouraged (24.64%); and self-management counseling (36.23%).

**Conclusions:**

gaps remain in Advanced Practice Nursing competencies. Academic training and the delivery of individualized care need to be strengthened. Effectively incorporating these competencies requires restructuring professional training and providing technical and scientific support.

## RESUMEN

**Objetivos:**

analizar las competencias de Enfermería de Práctica Avanzada (EPA) en promoción de la salud y prevención de enfermedades en consultas de salud de la mujer en la atención primaria de salud (APS).

**Métodos:**

estudio multicéntrico, descriptivo y transversal, con enfoque mixto, realizado en cuatro municipios brasileños. En 2022, 22 enfermeras realizaron 69 consultas grabadas; los datos se analizaron mediante estadística descriptiva y análisis de contenido.

**Resultados:**

se ofrecieron métodos anticonceptivos, orientaciones sobre infecciones de transmisión sexual, estrategias de promoción de la salud y cribado de enfermedades según protocolos, sin utilización de materiales educativos y sin acciones de prevención terciaria. Se fomentó el cambio de estilo de vida y la autogestión de la salud, aunque de forma limitada.

**Conclusiones:**

persisten brechas en las competencias de EPA en APS. Es necesario fortalecer la formación y el apoyo técnico y científico para consolidar una práctica más resolutiva y centrada en la mujer.

## INTRODUCTION

Over the years, many health systems worldwide have operated at the limits of their capacity, with overburdened services and limited financial resources, driven by the epidemiologic profile, changes in clinical conditions, growing population demand, and social and economic challenges. In this context, nursing plays a key role in strengthening health systems through safe, competent, and effective practice^(1,2)^.

Recently, new areas of nursing practice have emerged, such as the role of the advanced practice nurse-a professional with specialized knowledge, complex skills, and an expanded clinical scope, with strong potential to strengthen Primary Health Care (PHC)^(3,4)^. Advanced Practice Nursing (APN) is characterized by the integration and application of a broad range of theoretical and practical knowledge grounded in scientific evidence. This practice requires graduate-level nursing education, is shaped by the professional practice context, and is supported by specific certification and registration criteria established by regulatory bodies^(2,3)^.

In PHC, health promotion and disease prevention are core functions. Prevention is an early intervention based on knowledge of the natural history of disease and aims to stop its progression^(5,6)^. Nurses focus on epidemiologic and severity-related factors, prioritizing modifiable factors to reduce or control risks^(7)^. Prevention can be classified as primary, secondary, tertiary, and quaternary: primary prevention aims to reduce risks before clinical conditions develop; secondary prevention identifies early problems through timely detection; tertiary prevention seeks rehabilitation after health problems are established; and quaternary prevention aims to identify unnecessary treatments^(8,9)^.

Health promotion, in turn, is defined in the Ottawa Charter as a process that enables communities to improve their quality of life. It involves intersectoral actions engaging the health sector, government, and civil society^(10)^. Brazil’s National Health Promotion Policy (*Política Nacional de Promoção da Saúde*, PNPS) expands this concept by proposing individual and collective strategies implemented in coordination with the Health Care Network (*Rede de Atenção à Saúde*, RAS), fostering protection and social participation^(11)^. In PHC, women’s health care spans all stages of life and focuses on prevention, health promotion, and rehabilitation, as well as social, mental, and sexual dimensions^(12)^. Advanced practice nurses have achieved favorable results, especially in vulnerable areas, improving care and expanding coverage^(13)^. Nursing plays a central role in PHC, supporting health promotion and disease prevention, particularly in maternal health.

In light of this context, it is essential for nurses working in Brazilian PHC to discuss how to restructure their professional practice to align with Advanced Practice Nursing competencies. Achieving this goal requires robust, evidence-based technical and scientific support capable of effectively addressing health needs, as well as specific educational pathways recognized by the relevant regulatory bodies.

## OBJECTIVES

To analyze Advanced Practice Nursing competencies in health promotion and disease prevention during women’s health nursing consultations in Primary Health Care.

## METHODS

### Ethical aspects

The Research Ethics Committee of the participating institution approved the research project. All ethical principles governing research involving human participants were observed, in accordance with National Health Council Resolution 510/2016.

### Study design

This is a multicenter, descriptive, exploratory, cross-sectional study with a quantitative and qualitative approach.

### Study period

We analyzed 69 women’s health nursing consultations conducted between May 2 and July 29, 2022.

### Study setting

The study was conducted in Basic Health Units - BHU (*Unidades Básicas de Saúde*, UBS) in four Brazilian municipalities located in three regions of the country. Six units in the Northern Region (state of Amazonas - Municipality A), seven in the Northeastern Region (two in Alagoas [Municipality B] and five in Rio Grande do Norte [Municipality C]), and four in the Southeastern Region (state of São Paulo - Municipality D) were included. In total, 54 Family Health Strategy (*Estratégia Saúde da Família* - ESF) teams from 17 BHUs participated in the study. The units were selected for convenience, given the researchers’ geographic proximity and the feasibility of collaborating with local health services. This strategy was designed to represent different health system contexts in Brazil, enabling a comprehensive analysis of care practices in urban and rural areas with distinct socioeconomic and cultural conditions. The diversity of municipalities captured variation in access, organization, and the quality of care provided in BHUs, offering a broad view of the challenges faced in different health care settings across the country.

### Sample

The sample for this study consisted of data from recorded nursing consultations conducted as part of the project “Advanced Practice Nursing competencies in nursing consultations in primary health care: a multicenter study”. The study included 22 nurses and 69 women receiving care in BHUs across four Brazilian municipalities, identified as Municipalities A, B, C, and D, focusing on women’s health in PHC. Only participants aged 18 or older were included; therefore, no additional ethical procedures regarding minors were required.

### Inclusion criteria

For nurses, the inclusion criterion was working in PHC for at least one year. For women, the inclusion criterion was attending women’s health nursing consultations in BHUs. The data set included only fully recorded nursing consultations focused on women’s health.

### Exclusion criteria

We excluded professionals who were absent due to vacation or leave and women seen for reasons unrelated to women’s health, such as consultations addressing different life stages or non relevant clinical complaints, as well as consultations with technical problems in the recordings.

### Data collection

Data collection for the study titled “Advanced Practice Nursing competencies in nursing consultations in Primary Health Care: a multicenter study” was carried out in accordance with ethical principles for research. The project was submitted to local health authorities, and authorization to conduct data collection was obtained.

Nurses were approached during their routine work and were informed about the study procedures. Those who agreed to participate signed the Informed Consent Form (ICF), the Authorization Form for Use of Image and Voice, and completed a background information form. Data were collected using the REDCap platform, which ensured secure and confidential data storage.

Women were invited to participate in the study while waiting for their consultations in the waiting room; those who agreed also signed the ICF and the Authorization Form for Use of Image and Voice. For identification, they were given colored wristbands before the consultations started. The consultations were then recorded after the researchers left the room to avoid interfering with the care process.

### Instrument for assessing competencies in recorded consultations

To assess Advanced Practice Nursing competencies during the consultations, we developed an instrument based on the Prevention and Promotion domain of the Competency Profile, adapted from the tool developed by Cassiani et al. (2018). It was structured as a checklist with the following response options: 0 = Absent, 1 = Partially present, 2 = Present, and NA = Not applicable.

### Technological resources and data collection procedures

To ensure video quality, GoPro^®^ Hero9 cameras were configured according to local specifications (1080k x24, Superview, 1x lens). For consultations involving more than one professional, a mobile camera was attached to the nurse’s body to capture discussions between professionals. After each consultation, the researchers turned off the equipment and saved the recordings to an external hard drive and the Vimeo^®^ cloud platform, using security measures to protect data confidentiality.

### Data analysis

Data analysis combined quantitative and qualitative approaches, which were conducted separately but in a complementary way to deepen understanding of the phenomenon under study.

The quantitative component involved simple descriptive statistics, including the calculation of absolute and relative frequencies. We organized the data in Microsoft Excel spreadsheets and presented them in tables and figures to provide a clear view of the patterns observed.

The qualitative component followed the content analysis framework proposed by Bardin (2016), which includes three stages: pre-analysis; exploration of the material; and treatment of the results and interpretation. In the pre-analysis stage, we performed an initial reading of the material (transcripts of the recorded consultations), followed by the selection and organization of relevant content^(14)^.

We organized the transcripts in spreadsheets that included detailed descriptions of the scenes and the corresponding verbal evidence. The video recordings were examined in detail, and representative excerpts were selected according to whether the predefined competencies were present or absent, taking into account environmental, dialogic, and attitudinal aspects.

To support the organization and standardization of the qualitative analysis, we used a structured checklist with categorical variables (0 = Absent, 1 = Partially present, 2 = Present, and NA = Not applicable). This instrument guided the coding of the recording units and contributed to the development of thematic categories aligned with the study objectives. The lead researcher prepared the full transcription of all relevant material, ensuring data accuracy and integrity.

In the final stage, the data were interpreted in light of the theoretical framework, allowing us to draw inferences and identify patterns and categories. Thus, although conducted separately, the qualitative and quantitative analyses were complementary and yielded a broader and more consistent interpretation of the findings.

## RESULTS

We analyzed 69 women’s health nursing consultations conducted between May 2 and July 29, 2022, by 22 nurses across four municipalities: A (54.6%), B (9.0%), C (13.6%), and D (22.7%).

Most nurses (72.7%) were women, and 63.6% self-identified as parda (mixed race). The most frequent salary range was approximately seven times the minimum wage, reported by 45% of participants. Regarding professional experience, 45% had been in practice for more than 10 years, whereas 40% had been working in Family Health Strategy (Estratégia Saúde da Família, ESF) teams for less than one year. Approximately 59.1% held an additional job, and half of the sample (50%) reported commuting about 30 minutes to work. Most nurses worked in Basic Health Units with ESF teams (59.1%), and 54.5% reported not having access to a multidisciplinary team. In terms of professional motivation, 86.4% were satisfied with their work.

With respect to educational attainment, 54.5% had graduated from private institutions, and 95.5% had completed graduate education; 36.4% held a certificate diploma in Family Health and Public Health. In the previous year, 59.1% had taken continuing education courses, 63.6% of which focused on women’s health. In clinical practice, about 50% reported using nursing protocols, and 81.8% used the Primary Care Booklets (Cadernos de Atenção Básica) published by the Brazilian Ministry of Health.

Among the women, the mean age was 37 years, and 76.5% self-identified as parda (mixed race). Regarding marital status, 33.3% were never married, and 58% were not in a relationship. In terms of educational attainment, 20.3% had not completed elementary school, 59% had completed high school, and 10% had higher education. The unemployment rate was 46.4%, and 53.6% reported household incomes of up to 2 minimum wages. Most women lived in urban areas (83.3%), predominantly in Municipality A (59.4%).

Tables 1 and 2 present, respectively, the sociodemographic and professional profile of nurses working in municipalities A, B, C, and D (n = 22) and the sociodemographic profile of the women receiving care in these municipalities (n = 69).

**Table 1:** Sociodemographic and professional profile of nurses working in Municipalities A, B, C, and D, Brazil, 2025

NURSES		
Variables	n	%
**Years of experience as a nurse**		
1-5 years	3	13.6
6-10 years	9	40.9
> 10 years	10	45.5
**Team or service model in the unit**		
Family Health Strategy (ESF)	13	59.1
Mixed BHU	4	18.2
Traditional BHU	1	4.5
Rural BHU	4	18.2
**Years of experience as an ESF nurse**		
< 1 year	8	40
1-5 years	5	25
6-10 years	5	25
> 10 years	2	10
**Electronic health record**		
Yes	19	86.4
No	3	13.6
**Dedicated room for nursing consultations**		
Yes	17	77.3
No	5	22.7
**Difficulties in conducting consultations**		
Yes	15	68.2
No	7	31.8
**Standardized tools for the nursing process**		
Yes	7	31.8
No	15	68.2
**Nursing taxonomy**		
NANDA	5	22.7
CIPE	5	22.7
Other	4	18.2
None	8	36.4
**Type of undergraduate institution**		
Public	10	45.5
Private	12	54.5
**Graduate education**		
Yes	21	95.5
No	1	4.5
**Residency or graduate-level qualification**		
Certificate diploma in Family and Community Health, Collective Health, or Public Health	8	36.4
Certificate diploma in Mental Health or Psychiatry	2	9.1
Professional master’s degree	2	9.1
Academic master’s degree	1	4.5
Other (intensive care, obstetrics, emergency and urgent care, health promotion)	11	50
**Graduate studies**		
Completed a residency program or certificate diploma	12	54.5
Completed a master’s degree	3	13.6
Other studies	11	50
**Participation in courses in the past year**		
Women’s health	14	63.6
Child health	4	18.2
Health of older adults	3	13.6
Care for chronic conditions (hypertension/diabetes)	4	18.2
Acute care	3	13.6
Systematization of nursing care	4	18.2
Mental health	1	4.5
Nursing process	4	18.2
Other	4	18.2
**Number of courses (three categories)**		
None	5	22.7
1-2	13	59.1
> 3 courses	4	18.2
**Had classes on the nursing process**		
Yes	21	95.5
No	0	0
Do not know	1	4.5
**Use of nursing protocols in the unit**		
Yes	11	50
No	11	50
**Protocols used in women’s health**		
Primary Care Booklets (Cadernos de Atenção Básica) from the Brazilian Ministry of Health	18	81.8
Brazilian Ministry of Health protocol	16	72.7
State protocol	13	13.6
Protocols from other municipalities	2	9.1
Regional Nursing Council (Coren) protocol	8	36.4
Protocol issued by the municipal health department	11	50
Other	1	4.5

**Table 2:** Sociodemographic profile of women receiving care (N = 69) in Municipalities A, B, C, and D, Brazil, 2025

Women receiving care		
Characteristics	n	%
**Municipality**		
Municipality B	12	17.4
Municipality A	41	59.4
Municipality C	4	5.8
Municipality D	12	17.4
**Marital status**		
Common-law marriage	21	30.4
Never married	23	33.3
Divorced	2	2.9
Widowed	4	5.8
Married	19	27.5
**Number of children**		
No children	8	12
One child	18	26
Two children	12	17
Three children	15	22
Four or more children	16	23
**Has children**		
Yes	8	12
No	61	88
**Educational attainment**		
Elementary school or less	21	30
High school	41	59
Higher education	7	10
**Employment status**		
Currently working	27	39.1
Retired/receiving a pension	4	5.8
Not working or unemployed	38	55.1
**Occupation**		
Farmer	2	2.9
Domestic worker	18	26.1
Shopkeeper	1	1.4
Community Health Agent	1	1.4
Hairdresser/Manicurist	2	2.9
Seamstress	2	2.9
Intern	1	1.4
Teacher	1	1.4
Orthopedic immobilization technician	1	1.4
Caregiver for older adults	1	1.4
School monitor	1	1.4
Psychopedagogue	1	1.4
Salesperson	1	1.4
Coordinator	1	1.4
Nursing technician	2	2.9
Product customization specialist	1	1.4
Auxílio Brasil	1	1.4
Other	31	44.9
**Household income**		
No income	4	5.8
< 1 minimum wage	22	31.9
1-2 minimum wages	37	53.6
> 2 minimum wages	6	8.7
**Place of residence**		
Rural	6	8.8
Urban	57	83.8
Riverine area	5	7.3
**Live with others**		
Yes	6	8.7
No	63	91.3
**Age range of people in the household**		
0-11 years	42	39
12-18 years	19	28
19-59 years	49	71
≥ 60 years	8	12
**Was the consultation interrupted?**		
No	37	54
Yes	31	46

Prenatal care consultations accounted for 33% of all consultations, followed by visits for acute conditions (34%) and Pap smear tests (23%). The most common acute complaints included back, joint, or neck pain (36%), as well as problems such as insomnia (39%) and nervousness (28%). Regarding diagnoses, 22% of women had hypertension, 16% had diabetes, and 8.7% had anxiety or depression. Medical care was the most frequently sought type of care (67%), followed by nursing care (58%). The mean consultation length was 27 minutes, and 46% of consultations were interrupted.

In the health promotion domain, the competency “Participates in the development of health promotion programs” was demonstrated by the provision of contraceptive methods in 14.49% of visits, and only 30.30% of consultations addressed this topic. In prenatal care, folic acid and ferrous sulfate were prescribed in 86.2% of consultations. The competency “Selects, implements, and evaluates evidence-based strategies” was observed in 33.33% of consultations, and printed educational materials were not provided in 94.2% of visits. Disease screening was carried out in 47.16% of consultations, whereas tertiary and quaternary prevention actions were rare.

For disease screening, both subjective and objective data were collected in 55.37% of consultations, and clinical information was interpreted in 75.36%. Counseling to support the adoption of healthy lifestyle behaviors was documented in 24.64% of consultations. Assessment of women’s prior knowledge and development of an individualized care plan were recorded in 33.33% of consultations. Behavior-change training was provided in 40.58% of visits, and the same proportion included educational interventions to improve treatment adherence. The competency “Develops educational materials tailored to each woman” was not observed in any consultation.

**Figure 1:**
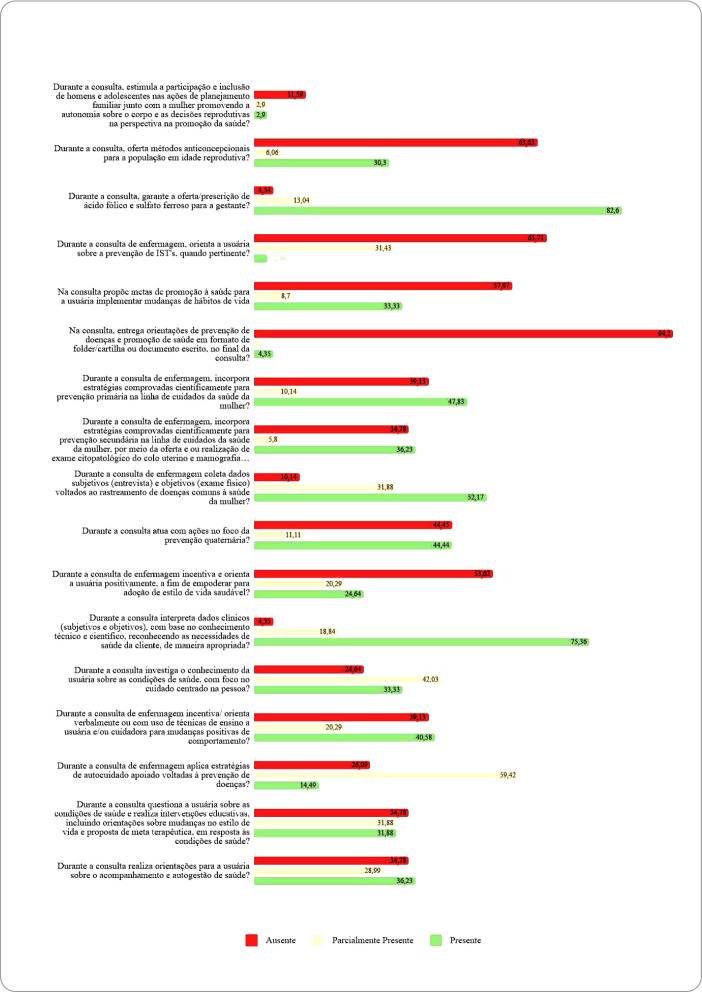
Description of Advanced Practice Nursing competencies observed in Primary Health Care consultations for health promotion and disease prevention, São Paulo, Brazil, 2025

In Figure 1, red, yellow, and green are used to make the results easier to interpret, following a familiar color scheme in graphs: red indicates competencies that are absent or areas requiring attention, yellow indicates competencies that are partially present or still developing, and green indicates competencies that are fully present or adequate.

## DISCUSSION

APN competencies in the health promotion and disease prevention domain within Brazilian PHC are still incipient. When they are present, nurses do not fully draw on them, which highlights the need for greater investment in strengthening their knowledge, skills, and attitudes.

In this study, the proportion of nurses with a certificate diploma (94.5%) was higher than that reported in previous research on advanced practice in PHC. In a study with PHC nurses from 14 Brazilian states, 75.95% of participants held a certificate diploma, whereas in a 2022 study with 161 nurses from Florianópolis, 56.6% had this level of training^(15,16)^. The proportion of nurses with a master’s degree was 13.6%, exceeding the figures reported in Báfica’s 2023 study, which examined the implementation of the APN role in PHC. Support from the Federal Nursing Council (Conselho Federal de Enfermagem, Cofen) is essential, particularly because relatively few nurses currently have access to professional master’s degree programs, which, unlike academic master’s degrees, are less widely available in nursing^(17-19)^.

Prenatal care was the most frequent reason for consultation (33%). Nurses have legal authority to provide prenatal nursing consultations, with autonomy to prescribe ferrous sulfate, folic acid, and medications for STIs according to syndromic protocols. They may also order tests, perform rapid tests, refer women to emergency obstetric care, provide guidance on immunobiologicals and vaccination, and conduct group-based health education activities. However, this prescribing competency is still not fully exercised in practice^(20)^.

The second most frequent reason for consultation (23%) was gynecological care, mainly Pap smear appointments. PHC nurses have autonomy to prescribe contraceptives and methods such as the IUD and Implanon during nursing consultations, contributing to reduced maternal mortality and broader access to care. Nevertheless, this competency is also underused by PHC nurses^(18,19)^.

Setting health promotion goals through lifestyle changes reveals a gap between what is advocated and what occurs in everyday nursing practice in PHC. Recent studies indicate that nurses often conflate health promotion with disease prevention, leading to a predominantly preventive focus centered on biological risk factors^(16,21-23)^. Although health promotion is widely presented as a core element of nursing work, care tends to be predominantly individual in practice, limiting the effectiveness of health promotion and making it less visible in routine consultations^(16,22,23)^.

Thus, APN-related competencies in health promotion and disease prevention in PHC remain underdeveloped and require investment in professional training. Despite the high proportion of nurses with a certificate diploma (94.5%) and a master’s degree (13.6%), access to professional master’s programs is still limited^(15,16)^.

Most consultations were for prenatal care (33%), followed by gynecologic consultations (23%), settings in which nurses are authorized to prescribe medications and perform procedures. However, these competencies are still not widely used in routine practice^(20)^. Health promotion is a particularly challenging area for nurses, who often confuse it with disease prevention^(21,24,25)^.

Although printed educational materials are valuable resources, the limited time nurses can devote to identifying women’s specific needs may undermine the effectiveness of health education. The absence or inappropriate use of these materials can substantially constrain the educational process, although it does not halt it altogether^(16)^. Primary prevention tends to be implemented only superficially, whereas secondary prevention is more highly valued by more experienced nurses^(26)^. Tertiary prevention was not addressed, and quaternary prevention faces barriers such as patients’ expectations and disease-centered marketing^(27,28)^.

Physical examination, a core component of the consultation, is often not performed comprehensively, especially during prenatal care^(29,30)^. Yet this competency needs strengthening, as it can significantly enhance the ability to address women’s health needs^(15)^.

Group activities have shown positive results for health promotion; however, individual consultations remain essential for providing more tailored care. The analysis of consultations indicates that the competency “encouraging and positively counseling women” was frequently observed. However, counseling focused on lifestyle changes still falls short of the desired effectiveness, indicating the need for further improvement^(31-33)^.

Lifestyle change, with an emphasis on empowerment and community participation, is more effective when supported by intersectoral actions. Nevertheless, the prevailing model remains largely biomedical, oriented toward specific groups and established diseases. Health promotion should instead prioritize quality of life and focus on preventing the onset of clinical conditions.

Although the ESF provides a favorable setting for patient-centered care, cultural resistance to change remains a challenge. The adoption of patient-centered approaches continues to face barriers within health system regulations^(34,35)^.

Patient participation in health care is fundamental for treatment success. In this study, however, the approach adopted during consultations was only partially implemented, pointing to the need for greater patient engagement in setting health goals. In this context, productivity pressures tend to reduce the time nurses have to listen to women, undermining the quality of care and health education. In addition, self-management-an important component of chronic condition management-was rarely addressed in PHC settings, despite its potential at this level of care^(36)^.

Self-management in health, as supported by the National Institute of Nursing Research (NINR), has been associated with reduced hospitalizations and better management of both acute and chronic conditions^(37-39)^.

### Study limitations

This study has limitations related to convenience sampling and the use of recorded consultations, which may cause discomfort for participants and introduce observer bias. The available scientific literature also constrains how these findings can be interpreted: Brazilian studies provide little detail on the practical role of ESF nurses in health promotion, and international literature does not clearly define the role of the advanced practice nurse in this context. The analysis was further limited by the absence of secondary data on care provided to older adults, genderand race-related issues, the health of rural and Indigenous workers, and other relevant aspects that could have enriched the analysis. Finally, the instrument was developed based on the adopted theoretical framework and the researchers’ expertise.

### Contributions to the field of nursing, health, and public policy

This study clarifies the competencies Primary Health Care nurses need to care for women, particularly APN-aligned competencies for health promotion and disease prevention. By analyzing and systematizing these competencies within nursing consultations, the study provides relevant evidence to support professional development and to inform the design of guidelines and public policies for the future implementation of APN in the Brazilian context. The findings also contribute to strengthening advanced practice and fostering the technical and scientific development of nursing in the country.

## FINAL CONSIDERATIONS

The findings of this study highlight gaps in the development of APN-related competencies in the health promotion and disease prevention domain during women’s health nursing consultations in PHC. The weaknesses identified in professional practice point to the need to strengthen these competencies from nursing education onward, with an emphasis on clinical practice and person-centered care.

To fully incorporate APN competencies in women’s health into clinical practice, it is essential to restructure both education and professional practice, supported by robust technical and scientific evidence and formal legal recognition by the relevant regulatory bodies. Implementing APN in Brazil will require investment in specialized, evidence-based training guided by women’s health needs to consolidate an expanded clinical practice that effectively addresses health promotion and disease prevention.

## Data Availability

The research data are available only upon request.

## References

[1] Gutiérrez-Rodríguez L, García-Mayor S, León-Campos Á, Gómez-González AJ, Pérez-Ardanaz B, Rodríguez-Gómez S (2022). Competency gradients in advanced practice nurses, specialist nurses, and registered nurses: a multicentre cross-sectional study. Int J Environ Res Public Health.

[2] International Council of Nurses (ICN) Nurse Practitioner (2014). Advanced Practice Nursing Network Country Profiles.

[3] Taylor A, Staruchowicz L. (2012). The experience and effectiveness of nurse practitioners in orthopaedic settings: a comprehensive systematic review. JBI Libr Syst Rev.

[4] Miranda MV, Rewa T, Leonello VM, Oliveira MAC. (2018). Advanced practice nursing: a possibility for Primary Health Care?. Rev Bras Enferm.

[5] Leavell H, Clark EG. (1976). Medicina preventiva.

[6] Ministério da Saúde (BR) (2013). Cadernos de Atenção Primária: Rastreamento.

[7] Soares JPR, Lourenço MP, Spigolon DN, Labegalini CMG, Costa MAR, Baldissera VDA. (2022). Promoção da saúde e prevenção de doenças: perspectivas de enfermeiros da atenção básica. Rev Enferm Cent-Oeste Min.

[8] Moll MD, Boff NN, Silva PSS, Siqueira TV, Ventura CAA. (2019). O enfermeiro na saúde da família e a promoção de saúde e prevenção de doenças. Enferm Foco.

[9] Westphal MF., Campos GWS (2009). Tratado de saúde coletiva.

[10] Ministério da Saúde (BR) (2002). As Cartas da Promoção da Saúde.

[11] Ministério da Saúde (BR) (2018). Política Nacional de Promoção da Saúde: PNPS.

[12] Mattos-Pimenta CA, Coca KP, Amorim MH, Belasco AG, Gabrielloni MC, Schirmer J. (2020). Prática Avançada em Enfermagem na Saúde da Mulher: formação em Mestrado Profissional. Acta Paul Enferm.

[13] Walker D, DeMaria L, Gonzalez-Hernandez D, Padron-Salas A, Romero-Alvarez M, Suarez L. (2013). Are all skilled birth attendants created equal? a cluster randomised controlled study of non-physician based obstetric care in primary health care clinics in Mexico. Midwifery.

[14] Bardin L. (2024). Análise de conteúdo.

[15] Cassiani SH, Aguirre-Boza F, Hoyos MC, Barreto MF, Morán L, Cerón MC (2018). Competências para a formação do enfermeiro de prática avançada para a atenção básica de saúde. Acta Paul Enferm.

[16] Minosso KC, Santos MB, Toso BRGO. (2024). Validation of the brazilian version of the modified scale for delineating advanced practice nursing roles. Rev Bras Enferm.

[17] Báfica AC. (2023). Prática avançada de enfermagem é uma realidade possível?.

[18] Gomes AM, Santos BM, Peres EM, Palha PF, Miranda MV, Silva MC (2024). Nota Técnica COFEN No. 01/2023: Práticas Avançadas de Enfermagem - PAE. Enferm Foco.

[19] Oliveira LS, Hermida PMV, Siqueira EF, Silva JCB, Thomas LS, Dalmolin IS. (2024). Evidências da inserção de dispositivo intrauterino por enfermeiros na Atenção Primária à Saúde: revisão integrativa. Rev Bras Enferm.

[20] Báfica AC, Gomes AM, Siqueira EF, Souza JM, Paese F, Belaver GM (2021). Atenção primária à saúde abrangente: ampliando acesso para uma enfermagem forte e resolutiva. Enferm Foco.

[21] Ministério da Saúde (BR) (2012). Atenção ao pré-natal de baixo risco.

[22] Brandão LGVA, Teixeira CC, Afonso TC, Amaral RT, Bezerra ALQ. (2019). O sentido do trabalho naAtenção Primária à Saúde. REAS.

[23] Silva NCDCD, Mekaro KS, Santos RIO, Uehara SCDSA. (2020). Knowledge and health promotion practice of Family Health Strategy nurses. Rev Bras Enferm.

[24] Mascarenhas NB, Melo CMM, Fagundes NC. (2012). Produção do conhecimento sobre promoção da saúde e prática da enfermeira na Atenção Primária. Rev Bras Enferm.

[25] Tavares RE, Tocantins FR. (2015). Nursing actions in primary care and the control of diseases preventable through vaccines. Rev Bras Enferm.

[26] Ministério da Saúde (BR) (2013). Institui a Política Nacional de Educação Popular em Saúde no SUS.

[27] Gualdezi LF. (2021). Competências do enfermeiro em práticas avançadas de enfermagem na Atenção Primária à Saúde.

[28] Schopf K, Vendruscolo C, Silva CB, Geremia DS, Souza AL, Angonese LL. (2022). Prevenção Quaternária na Atenção Primária à Saúde. Esc Anna Nery.

[29] Norman AH, Tesser CD. (2009). Prevenção quaternária na atenção primária à saúde: uma necessidade do SUS. Cad Saúde Pública.

[30] Conselho Federal de Enfermagem (Cofen) (2024). Resolução nº 736, de 17 de janeiro de 2024.

[31] Lima SGS, Spagnuolo RS, Juliani CMCM, Colichi RMB. (2022). Nursing consultation in the Family Health Strategy and the nurse’s perception. Rev Bras Enferm.

[32] Silva NCC, Mekaro KS, Santos RIO, André-Uehara SCS. (2020). Knowledge and health promotion practice of Family Health Strategy nurses. Rev Bras Enferm.

[33] Sacramento RC, Vendruscolo C, Silva CB, Metelski FK, Trindade LL, Adamy EK. (2023). Dimensões assistenciais do trabalho do enfermeiro. Esc Anna Nery.

[34] Heidemann ITSB, Juvinyà-Canal D, Durand MK, Reig-Garcia G, Correa SM, Araújo LMC (2023). Práticas de promoção da saúde na atenção primária: comparativo entre Florianópolis-Brasil e Girona-Espanha. Texto Contexto Enferm.

[35] Carvalho PR, Ferraz ESD, Teixeira CC, Machado VB, Bezerra ALQ, Paranaguá TTB. (2021). Patient participation in care safety: Primary Health Care professionals’ perception. Rev Bras Enferm.

[36] Ventura F, Moreira IMPB, Raposo V, Queirós PJP, Mendes A. (2022). A prática centrada na pessoa: da idiossincrasia do cuidar à inovação em saúde. Cad Saúde Pública.

[37] Ferreira DS, Ramos FRS, Teixeira E. (2021). Nurses’ educational practices in Family Health Strategy. Rev Bras Enferm.

[38] Otter CEM, Keers JC, Reker C, Smit J, Schoonhoven L, de Man-van Ginkel JM. (2022). How nurses support self-management of hospitalized patients through verbal communication: a qualitative study. BMC Nurs.

[39] Allory E, Scheer J, Andrade V, Chan K, Brousselle A. (2024). Characteristics of self-management education and support programmes for people with chronic diseases delivered by primary care teams: a rapid review. BMC Prim Care.

