# Supplementary material for: Health promotion and disease prevention: Advanced Practice Nursing competencies in women’s health consultations
Source: Rev Bras Enferm. 2026 Jul 27;79:e20250064. doi: 10.1590/0034-7167-2025-0064 (PMC13411323; doi:10.1590/0034-7167-2025-0064)
Supplement: Translation

# Translation: Promoção da saúde e prevenção de doenças: competências de Práticas Avançadas de Enfermagem nas consultas de saúde das mulheres

**DOI:** 10.1590/0034-7167-2025-0064pt

**Authors:** Jaqueline Martins Ramos, Daiana Bonfim, Andrea Liliana Vesga-Varela, Manoel Vieira de Miranda

**Affiliations:** I Hospital Israelita Albert Einstein, Faculdade Israelita de Ciências da Saúde Albert Einstein. São Paulo, São Paulo, Brasil; II Hospital Israelita Albert Einstein. São Paulo, São Paulo, Brasil; III Universidade de São Paulo. São Paulo, São Paulo, Brasil

**Keywords:** Prática Avançada de Enfermagem, Enfermagem de Atenção Primária, Atenção Primária à, Saúde, Promoção da Saúde, Saúde da Mulher.

## Abstract

**Objetivos:**

analisar as competências de Práticas Avançadas de Enfermagem no domínio da promoção da saúde e prevenção de doenças em consultas voltadas à saúde das mulheres na Atenção Primária.

**Métodos:**

estudo multicêntrico, descritivo, exploratório, transversal, com abordagem quantitativo-qualitativa, em quatro municípios brasileiros do Norte, Nordeste e Sudeste. Em 2022 (maio-julho), 22 enfermeiros conduziram 69 consultas gravadas - dados organizados no Excel e analisados com estatísticas descritivas e análise de conteúdo.

**Resultados:**

ofertaram-se métodos contraceptivos (30,3% das consultas); orientações sobre infecções sexualmente transmissíveis (2,86%); estratégias de promoção da saúde (49,25%), sem materiais educativos impressos; rastreamento de doenças conforme protocolos (47,16%); ações de prevenção terciária ausentes; incentivos à mudança de estilo de vida (24,64%) e orientações sobre autogestão (36,23%).

**Conclusões:**

há lacunas nas competências de Práticas Avançadas de Enfermagem. Necessita-se aprimoramento desde a formação acadêmica e qualificação do cuidado individualizado. Incorporar efetivamente essas competências requer reestruturação da formação profissional e suporte técnico-científico.

## INTRODUÇÃO

Ao longo dos anos, diversos sistemas de saúde pelo mundo têm operado no limite de sua capacidade, com sobrecarga de atendimento e escassez de recursos financeiros, decorrentes do cenário epidemiológico, de variações das condições clínicas, de aumento de demanda populacional e de condições sociais e econômicas. Diante desse cenário, o protagonismo da enfermagem torna se fundamental para aprimorar o sistema de saúde, por meio de uma atuação segura, qualificada e efetiva^(1,2)^.

Recentemente, têm surgido novos ramos de atuação na enfermagem, como o do enfermeiro de práticas avançadas (EPA) - profissional detentor de conhecimento especializado, habilidades complexas e escopo clínico ampliado, com grande potencial de qualificar a Atenção Primária à Saúde (APS)^(3,4)^. A Prática Avançada de Enfermagem (PAE) caracteriza-se pela integração e aplicação de um amplo conjunto de conhecimentos teóricos e práticos, fundamentado em evidências científicas. Essa prática resulta de formação em nível de pós-graduação em Enfermagem, sendo moldada conforme o contexto de atuação profissional e respaldada por critérios específicos de certificação e registro, definidos por órgãos reguladores^(2,3)^.

Na APS, a promoção da saúde e a prevenção de doenças são pilares fundamentais. A prevenção, enquanto intervenção precoce fundamentada no conhecimento da história natural da enfermidade, tem como objetivo impedir sua progressão^(5,6)^. A atuação do enfermeiro centra-se em fatores epidemiológicos e de gravidade, priorizando os fatores modificáveis para reduzir ou controlar os riscos^(7)^. A prevenção pode ser classificada em primária, secundária, terciária e quaternária: a primária visa reduzir riscos antes da instalação de condições clínicas; a secundária identifica problemas iniciais com detecção precoce; a terciária busca reabilitação após a instalação de problemas de saúde; e a quaternária detecta tratamentos desnecessários^(8,9)^.

Já a promoção da saúde, conforme a Carta de Ottawa, é um processo de capacitação da comunidade para melhorar a qualidade de vida. Isso envolve ações intersetoriais com participação do setor saúde, governo e sociedade civil^(10)^. A Política Nacional de Promoção da saúde (PNPS) amplia esse conceito, propondo estratégias individuais e coletivas articuladas com a Rede de Atenção à Saúde (RAS), favorecendo a proteção e participação social^(11)^. Na APS, a assistência à saúde das mulheres abrange cuidados em todas as fases da vida, com foco na prevenção, promoção e reabilitação, bem como em aspectos sociais, mentais e sexuais^(12)^. A atuação do EPA tem mostrado resultados favoráveis, especialmente em zonas vulneráveis, com melhorias no atendimento e aumento da cobertura^(13)^. A enfermagem desempenha papel central na APS, favorecendo a promoção da saúde e a prevenção de doenças, especialmente na saúde materna.

Diante do exposto e do cenário atual de saúde apresentado, torna-se basilar que enfermeiros da APS brasileira discutam a reestruturação da prática profissional voltada às competências de Práticas Avançadas de Enfermagem. Para que seja possível, é essencial o aporte técnico científico baseado em evidências e com capacidade altamente resolutiva, além de formação específica com reconhecimento pelos órgãos competentes.

## OBJETIVOS

Analisar as competências de Práticas Avançadas de Enfermagem na promoção da saúde e prevenção de doenças nas consultas de enfermagem voltadas à saúde das mulheres na Atenção Primária à Saúde.

## MÉTODOS

### Aspectos éticos

O projeto de pesquisa foi aprovado pelo Comitê de Ética em Pesquisa (CEP) da instituição envolvida. Foram observados todos os preceitos éticos que regulamentam pesquisas com seres humanos, em conformidade com a Resolução 510/2016 do Conselho Nacional de Saúde.

### Desenho do estudo

Trata-se de um estudo multicêntrico, descritivo e exploratório, de natureza transversal, com abordagem quantitativo-qualitativa.

### Período

Foram analisadas 69 consultas de enfermagem na área de saúde das mulheres, realizadas entre 2 de maio e 29 de julho de 2022.

### Local de estudo

A seleção dos cenários de pesquisa contemplou Unidades Básicas de Saúde (UBS) de quatro municípios brasileiros, distribuídas em três regiões do país. Foram incluídas seis unidades de APS na Região Norte (estado do Amazonas - Município A), sete unidades na Região Nordeste (sendo duas em Alagoas [Município B] e cinco no Rio Grande do Norte [Município C]) e quatro unidades na Região Sudeste (estado de São Paulo - Município D). No total, participaram do estudo 54 equipes da Estratégia Saúde da Família (ESF), distribuídas em 17 unidades de APS. A escolha foi realizada por conveniência, considerando a proximidade geográfica das pesquisadoras e a facilidade de articulação com os serviços de saúde locais. A seleção foi estratégica para representar diferentes contextos de saúde no Brasil, permitindo uma análise abrangente das práticas de saúde em áreas urbanas e rurais, com realidades socioeconômicas e culturais distintas. A diversidade dos municípios refletiu as variações no acesso, organização e qualidade da atenção à saúde nas UBS, proporcionando uma visão ampla dos desafios enfrentados em diferentes cenários de saúde no país.

### Amostra

A amostra deste estudo foi composta por dados de consultas de enfermagem gravadas do projeto “Competências de Prática Avançada nas consultas de enfermagem na Atenção Primária à Saúde: um estudo multicêntrico”. A pesquisa envolveu 22 profissionais de enfermagem e 69 usuárias atendidas nas UBS de quatro municípios brasileiros, identificados como Municípios A, B, C e D, com foco na saúde das mulheres na APS. A amostra da pesquisa foi composta exclusivamente por participantes maiores de 18 anos, não sendo necessária a adoção de procedimentos éticos adicionais voltados à participação de menores.

### Critérios de inclusão

Para os enfermeiros, o critério de inclusão foi a atuação na APS por pelo menos um ano. Para as usuárias, o critério foi o atendimento em consultas de enfermagem voltadas à saúde das mulheres nas UBS. O critério de seleção do banco de dados foi a gravação completa de consultas de enfermagem relacionadas à saúde das mulheres.

### Critérios de exclusão

Foram excluídos os profissionais ausentes por férias ou afastamento e as usuárias atendidas por motivos não relacionados à saúde das mulheres, com consultas em ciclos de vida diferentes, queixas clínicas não pertinentes e falhas técnicas nas gravações.

### Coleta de dados

A coleta de dados do estudo intitulado “Competências de prática avançada nas consultas de enfermagem na Atenção Primária à Saúde: um estudo multicêntrico” foi conduzida em conformidade com os princípios éticos da pesquisa. O projeto foi submetido à gestão local, sendo obtida a autorização para realizar a coleta de dados.

Os profissionais enfermeiros foram abordados durante o exercício de suas atividades laborais, sendo-lhes apresentada a metodologia do estudo. Aqueles que consentiram em participar assinaram o Termo de Consentimento Livre e Esclarecido (TCLE), o Termo de Autorização para Uso de Imagem e Voz, bem como o instrumento de caracterização. A coleta dos dados foi efetuada por meio da plataforma REDCap, assegurando o armazenamento seguro e confidencial das informações.

As usuárias foram convidadas a integrar a pesquisa enquanto aguardavam atendimento na sala de espera, ocasião em que também assinaram o TCLE e o Termo de Autorização para Uso de Imagem e Voz. Para a identificação, receberam pulseiras coloridas antes do início das consultas. Na sequência, as consultas foram gravadas após os pesquisadores retirarem-se do ambiente, para garantir a não interferência no processo assistencial.

### Instrumento para coleta de competências em consultas gravadas

Para avaliar as competências de Prática Avançada de Enfermagem nas consultas, foi desenvolvido um instrumento baseado no domínio Prevenção e Promoção do Perfil de Competências, de uma ferramenta elaborada por Cassiani et al. (2018). Ele foi estruturado na forma de *checklist* com as seguintes opções de resposta: 0 = Ausente, 1 = Parcialmente presente, 2 = Presente e NA = Não se aplica.

### Recursos tecnológicos e procedimentos de coleta

Para garantir a qualidade da captação de vídeo, as câmeras GoPro^®^ Hero9 foram ajustadas conforme as especificações locais (1080k x24, Superview, lente 1x). Em casos de interconsulta, uma câmera móvel foi fixada ao corpo do enfermeiro para registrar as discussões entre profissionais. Após o atendimento, os pesquisadores desligaram os equipamentos e armazenaram os dados em HD externo e no software de nuvem Vimeo^®^, adotando medidas de segurança para preservar a confidencialidade das informações.

### Análise de dados

A análise dos dados foi realizada por meio de abordagens qualitativa e quantitativa, conduzidas de forma separada, mas complementar, a fim de ampliar a compreensão do fenômeno investigado.

A abordagem quantitativa consistiu na aplicação de estatística descritiva simples, com apuração de frequências absolutas e relativas. Os dados foram organizados em planilhas eletrônicas (Microsoft Excel) e apresentados em gráficos e tabelas, permitindo a visualização objetiva dos padrões observados.

Já a abordagem qualitativa foi conduzida com base no referencial metodológico da Análise de Conteúdo proposta por Bardin (2016), constituída por três etapas: pré-análise; exploração do material; e tratamento dos resultados obtidos e interpretação. Na fase de pré-análise, realizou-se a leitura flutuante das transcrições das consultas gravadas, seguida pela seleção e organização dos materiais relevantes^(14)^.

As transcrições foram sistematizadas em planilhas eletrônicas, contendo a descrição detalhada das cenas e as respectivas evidências verbais. O conteúdo das filmagens foi criteriosamente examinado, e trechos representativos foram selecionados conforme a ocorrência (ou não) de competências previamente definidas, considerando aspectos ambientais, dialógicos e atitudinais.

Para auxiliar a sistematização e padronização da análise qualitativa, utilizou-se um *checklist* estruturado com variáveis categóricas (0 = ausente, 1 = parcialmente presente, 2 = presente e NA = não se aplica). Esse instrumento orientou a codificação das unidades de registro e contribuiu para a construção das categorias temáticas alinhadas aos objetivos do estudo. A transcrição integral do material considerado relevante foi realizada pelo pesquisador principal, garantindo a fidelidade e integridade dos dados.

Na etapa final, os dados foram interpretados à luz do referencial teórico, permitindo fazer inferências bem como identificar padrões e categorias. Dessa forma, embora conduzidas separadamente, as análises qualitativa e quantitativa foram complementares, resultando em uma interpretação ampliada e consistente dos achados.

## RESULTADOS

Foram analisadas 69 consultas de saúde da mulher, realizadas entre 2 de maio e 29 de julho de 2022, por 22 enfermeiros distribuídos em quatro municípios: A (54,6%), B (9%), C (13,6%) e D (22,7%).

A maior parte dos enfermeiros (72,7%) eram do sexo feminino, e 63,6% declararam-se pardos. A faixa salarial média correspondia a sete salários mínimos, com 45% dos participantes inseridos nessa categoria. Quanto ao tempo de experiência profissional, 45% tinham mais de dez anos de atuação, enquanto 40% estavam vinculados à Estratégia Saúde da Família há menos de um ano. Aproximadamente 59,1% dos enfermeiros mantinham vínculo empregatício adicional, e metade da amostra (50%) gastava cerca de 30 minutos para se deslocar até o local de trabalho. A maioria exercia suas funções em unidades com ESF (59,1%), e 54,5% relataram não contar com apoio multiprofissional. No que tange à motivação profissional, 86,4% manifestaram satisfação com o trabalho realizado.

Quanto à formação acadêmica, 54,5% dos profissionais graduaram-se em instituições privadas, e 95,5% tinham pós-graduação, com 36,4% especializados em Saúde da Família e Saúde Pública. No período de um ano, 59,1% participaram de cursos de atualização, dos quais 63,6% eram sobre saúde da mulher. Em relação à prática clínica, cerca de 50% utilizavam protocolos de enfermagem, e 81,8% empregavam os Cadernos de Atenção Básica do Ministério da Saúde.

Já no tocante às usuárias, a média de idade foi de 37 anos, sendo 76,5% autodeclaradas pardas. No que se refere ao estado civil, 33,3% eram solteiras, e 58% não tinham parceiros. Quanto à escolaridade, 20,3% apresentavam ensino fundamental incompleto; 59%, ensino médio completo; e 10%, ensino superior. O índice de desemprego foi de 46,4%, e 53,6% referiram renda familiar de até dois salários mínimos. A maioria residia em áreas urbanas (83,3%), com maior concentração no Município A (59,4%).

As Tabelas 1 e 2 apresentam, respectivamente, o perfil sociodemográfico e profissional das enfermeiras atuantes nos municípios A, B, C e D (n = 22) e o perfil sociodemográfico das usuárias atendidas nesses municípios (n = 69).

**Tabela 1:** Perfil sociodemográfico e profissional de enfermeiras atuantes nos Municípios A, B, C e D, Brasil, 2025

ENFERMEIROS		
Variáveis	n	%
**Tempo de experiência como enfermeiro**		
Entre 1 e 5 anos	3	13,6
Entre 6 e 10 anos	9	40,9
> 10 anos	10	45,5
**Tipo de equipe na unidade**		
Estratégia Saúde da Família	13	59,1
UBS mista	4	18,2
UBS tradicional	1	4,5
UBS rural	4	18,2
**Tempo de experiência como enfermeiro da ESF**		
< 1 ano	8	40
Entre 1 e 5 anos	5	25
Entre 6 e 10 anos	5	25
> 10 anos	2	10
**Prontuário eletrônico**		
Sim	19	86,4
Não	3	13,6
**Existe sala exclusiva para consulta de enfermagem?**		
Sim	17	77,3
Não	5	22,7
**Existem dificuldades para realização de consulta?**		
Sim	15	68,2
Não	7	31,8
**Existem instrumentos padronizados do processo de enfermagem?**		
Sim	7	31,8
Não	15	68,2
**Taxonomia de enfermagem**		
NANDA	5	22,7
CIPE	5	22,7
Outros	4	18,2
Nenhuma	8	36,4
**Tipo de universidade da graduação**		
Pública	10	45,5
Privada	12	54,5
**Possui pós-graduação?**		
Sim	21	95,5
Não	1	4,5
**Residência ou especialização**		
Espec. Saúde da Família e Comunidade, Saúde Coletiva ou Saúde Pública	8	36,4
Espec. Saúde Mental ou Psiquiatria	2	9,1
Mestrado profissional	2	9,1
Mestrado acadêmico	1	4,5
Outro (terapia intensiva, obstetrícia, urgências e emergência, promoção da saúde)	11	50
**Estudos de pós-graduação**		
Fez residência ou especialização	12	54,5
Fez mestrado	3	13,6
Outros estudos	11	50
**Participação em cursos no último ano**		
Saúde da mulher	14	63,6
Saúde da criança	4	18,2
Saúde do idoso	3	13,6
Cuidado a crônicos (hipertensão/diabetes)	4	18,2
Atendimento de casos agudos	3	13,6
Sistematização da assistência de enfermagem	4	18,2
Saúde mental	1	4,5
Processo de enfermagem	4	18,2
Outros	4	18,2
**Número de cursos (três categorias)**		
Nenhum	5	22,7
Entre 1 e 2	13	59,1
> 3 cursos	4	18,2
**Teve aulas sobre o processo de enfermagem?**		
Sim	21	95,5
Não	0	0
Não sei	1	4,5
**Na sua unidade, usam protocolos de enfermagem?**		
Sim	11	50
Não	11	50
**Protocolos usados na saúde da mulher**		
Caderno de Atenção Básica do Ministério da Saúde	18	81,8
Protocolo Ministério da Saúde	16	72,7
Protocolo do estado	13	13,6
Protocolo de outros municípios	2	9,1
Protocolo do Coren	8	36,4
Protocolo da sua secretaria municipal de saúde	11	50
Outros	1	4,5

**Tabela 2:** Perfil sociodemográfico de usuárias (N = 69) nos Municípios A, B, C e D, Brasil, 2025

Usuárias		
Características	n	%
**Município**		
Município B	12	17,4
Município A	41	59,4
Município C	4	5,8
Município D	12	17,4
**Estado civil**		
União estável	21	30,4
Solteiro(a)	23	33,3
Divorciado(a)	2	2,9
Viúvo(a)	4	5,8
Casado(a)	19	27,5
**Número de filhos**		
Sem filhos	8	12
Um filho	18	26
Dois filhos	12	17
Três filhos	15	22
Quatro ou mais filhos	16	23
**Tem filhos?**		
Sim	8	12
Não	61	88
**Nível de escolaridade**		
Até ensino fundamental	21	30
Até ensino médio	41	59
Até ensino superior	7	10
**Trabalho**		
Trabalhando	27	39,1
Aposentado/pensionista	4	5,8
Não trabalha ou desempregado	38	55,1
**Profissão**		
Agricultora	2	2,9
Doméstica	18	26,1
Comerciante	1	1,4
Agente Comunitária de Saúde	1	1,4
Cabelereira/Manicure	2	2,9
Costureira	2	2,9
Estagiária	1	1,4
Professora	1	1,4
Técnica de imobilização ortopédica	1	1,4
Cuidadora de idoso	1	1,4
Monitora escolar	1	1,4
Psicopedagoga	1	1,4
Vendedora	1	1,4
Coordenadora	1	1,4
Técnica de Enfermagem	2	2,9
Customizador de produtos	1	1,4
Auxílio Brasil	1	1,4
Outros	31	44,9
**Renda familiar**		
Sem renda	4	5,8
< 1 salário mínimo	22	31,9
Entre 1 e 2 salários mínimos	37	53,6
> 2 salários mínimos	6	8,7
**Local de moradia**		
Rural	6	8,8
Urbana	57	83,8
Ribeirinha	5	7,3
**Mora acompanhado**		
Sim	6	8,7
Não	63	91,3
**Faixa de idade das pessoas da casa**		
Entre 0 e 11 anos	42	39
Entre 12 e 18 anos	19	28
Entre 19 e 59 anos	49	71
≥ 60 anos	8	12
**A consulta foi interrompida?**		
Não	37	54
Sim	31	46

As consultas de pré-natal representaram 33% do total, configurando-se como as mais frequentes, seguidas pelos atendimentos relacionados a eventos agudos (34%) e aos exames de Papanicolau (23%). As queixas agudas mais comuns incluíram dores na coluna, articulações ou pescoço (36%), além de dificuldades como insônia (39%) e nervosismo (28%). Em relação aos diagnósticos, 22% dos pacientes apresentavam hipertensão; 16%, diabetes; e 8,7%, ansiedade ou depressão. A busca por atendimento médico foi a mais frequente (67%), seguida da procura por enfermeiros (58%). A duração média das consultas foi de 27 minutos, sendo que 46% delas foram interrompidas.

No eixo de promoção da saúde, a competência “Participa do desenvolvimento de programas de promoção da saúde” foi evidenciada pela oferta de métodos contraceptivos em 14,49% dos atendimentos, e apenas 30,30% das consultas abordaram o tema. No pré-natal, a prescrição de ácido fólico e sulfato ferroso ocorreu em 86,2%. A competência “Seleciona, implementa e avalia estratégias baseadas em evidências” foi observada em 33,33% das consultas, e não houve entrega de materiais educativos em 94,2% dos atendimentos. O rastreamento de doenças ocorreu em 47,16% das consultas, enquanto ações de prevenção terciária e quaternária foram raras.

Na coleta de dados para rastreamento de doenças, dados subjetivos e objetivos estiveram presentes em 55,37% das consultas, e a interpretação das informações técnicas se deu em 75,36%. Quanto ao empoderamento para adoção de estilo de vida saudável, a orientação esteve presente em 24,64% das consultas. A investigação do conhecimento da usuária e o plano de cuidados individualizado foram realizados em 33,33% das consultas. O treinamento para mudança de comportamento ocorreu em 40,58% dos atendimentos, e a mesma taxa foi observada no tocante às intervenções educativas sobre adesão ao tratamento. A competência de desenvolver materiais educativos específicos para a usuária não constou em nenhum atendimento.

Na Figura 1, as cores vermelho, amarelo e verde foram utilizadas para facilitar a visualização dos resultados, seguindo uma convenção comum em gráficos: vermelho representa competências ausentes ou áreas que requerem atenção, amarelo indica competências parcialmente presentes ou em desenvolvimento, e verde sinaliza competências plenamente presentes ou adequadas.

**Figura 1:** Descrição das competências de Práticas Avançadas de Enfermagem presentes em consultas da Atenção Primária à Saúde para a promoção da saúde e prevenção de doenças, São Paulo, Brasil, 2025

## DISCUSSÃO

As competências esperadas pelo EPA no domínio de promoção da saúde e prevenção de doenças no contexto da APS brasileira ainda são incipientes. Quando presentes, não são completamente exploradas pelos enfermeiros, o que evidencia a necessidade de maior investimento na qualificação de seus conhecimentos, habilidades e atitudes.

Os participantes deste estudo apresentaram uma taxa de especialização superior à observada em pesquisas anteriores sobre práticas avançadas na APS (94,5%). Em estudo com enfermeiros da APS de 14 estados brasileiros, 75,95% dos participantes possuíam especialização, enquanto em pesquisa de 2022 com 161 enfermeiros de Florianópolis, 56,6% tinham nível de e^(15,16)^. O nível de mestrado entre os profissionais foi de 13,6%, superando os dados do estudo de Báfica (2023), que discutiu a implementação do cargo de EPA na APS. O incentivo do Cofen é crucial, especialmente porque ainda são poucos os profissionais com acesso à formação em mestrado profissional - modalidade que, diferentemente do mestrado acadêmico, está menos disseminada entre os enfermeiros^(17-19)^.

A maior demanda de atendimentos foi para a realização de pré-natal (33%). O enfermeiro tem respaldo legal para realizar consultas de enfermagem à gestante, com autonomia para prescrição de sulfato ferroso, ácido fólico e medicamentos para ISTs conforme protocolo sindrômico. Além disso, pode solicitar exames, realizar testes rápidos, encaminhar a emergências obstétricas, orientar sobre imunobiológicos e vacinação e conduzir atendimentos coletivos de educação em saúde. No entanto, a competência prescritiva ainda não é amplamente exercida^(20)^.

A segunda maior demanda de atendimentos (23%) foi para a consulta ginecológica, com ênfase na realização do exame Papanicolau. O enfermeiro da APS tem autonomia para prescrever anticoncepcionais e métodos como o DIU/Implanon dentro da consulta de enfermagem, contribuindo para a redução da mortalidade materna e ampliando o acesso aos cuidados. Todavia, essa competência também é pouco usada pelos enfermeiros da APS^(18,19)^.

As metas de promoção da saúde por meio de mudanças nos hábitos de vida evidenciam uma contradição entre o discurso e a prática de enfermagem na APS. Estudos recentes indicam que os enfermeiros frequentemente confundem promoção da saúde com prevenção de doenças, resultando em um enfoque predominante na prevenção, com ênfase em aspectos biológicos^(16,21-23)^. Embora a promoção da saúde seja amplamente divulgada como um elemento importante no trabalho dos enfermeiros, a realidade observada nos serviços é que o atendimento tende a ser proeminentemente individual, o que limita a efetividade da promoção da saúde e torna sua presença, no contexto do atendimento, pouco evidenciada^(16,22,23)^.

As competências relacionadas à promoção da saúde e prevenção de doenças na APS permanecem em estágio inicial, exigindo investimentos em capacitação profissional. Apesar da alta taxa de especialização (94,5%) e mestrado (13,6%) entre os enfermeiros, o acesso à formação em mestrado profissional ainda é limitado^(15,16)^.

O maior volume de atendimentos relacionou-se ao pré-natal (33%), seguido pela consulta ginecológica (23%), nos quais os enfermeiros têm autonomia para prescrever e realizar procedimentos. Porém, essas competências ainda não são plenamente praticadas^(20)^. A promoção da saúde é uma área desafiadora para os enfermeiros, que frequentemente a confundem com a prevenção de doenças^(21,24,25)^.

Embora os materiais educativos impressos constituam recursos relevantes, a falta de tempo dedicado pelos enfermeiros para identificar as necessidades específicas dos usuários pode comprometer a eficácia da educação em saúde. A ausência e a utilização inadequada desses materiais podem limitar significativamente o processo educativo, sem, entretanto, anulá-lo^(16)^. A prevenção primária é realizada de forma inicial, enquanto a prevenção secundária é mais valorizada por enfermeiros mais experientes^(26)^. A prevenção terciária não foi abordada, e a prevenção quaternária enfrenta barreiras como as expectativas dos usuários e a ênfase no marketing centrado na doença^(27,28)^.

O exame físico, componente fundamental da consulta, frequentemente não é realizado de maneira completa, sobretudo nas consultas de pré-natal^(29,30)^. Entretanto, esta é uma competência relevante a ser aprimorada, já que pode contribuir consideravelmente para a resolutividade do cuidado^(15)^.

As atividades em grupo apresentam resultados positivos na promoção da saúde; contudo, a consulta individual é imprescindível para a oferta de um cuidado mais específico. A análise das consultas indica que a competência de “incentivar e orientar a usuária de forma positiva” é significativa, porém as orientações voltadas à mudança do estilo de vida ainda não alcançam a efetividade desejada, revelando que é necessário o aprimoramento^(31-33)^.

A mudança de estilo de vida, com ênfase na emancipação e participação popular, é mais eficaz quando realizada intersetorialmente, porém o modelo ainda é predominantemente biomédico, voltado para grupos específicos e doenças já instaladas. Em vez disso, a promoção da saúde deveria focalizar a qualidade de vida e anteceder a condição clínica diagnóstica.

Embora a ESF seja um ambiente propício ao cuidado centrado no paciente, a resistência à mudança de cultura é um desafio. A adoção de abordagens centradas no paciente ainda encontra obstáculos dentro das regulamentações do sistema de saúde^(34,35)^.

A participação do usuário no processo de saúde é fundamental para o sucesso do tratamento. No entanto, a abordagem durante as consultas foi parcialmente implementada, indicando a necessidade de maior envolvimento dos usuários na definição de metas de saúde. Nesse contexto, a pressão pela produtividade tende a reduzir o tempo de escuta do profissional, o que prejudica a qualidade do atendimento e a educação em saúde. Além disso, a autogestão, importante no manejo de condições crônicas, foi pouco trabalhada na APS, mesmo diante de seu potencial nesse nível de atenção^(36)^.

A autogestão em saúde, como apoiado pelo *National Institute of Nursing Research* (NINR), tem mostrado benefícios na redução de hospitalizações e melhora na gestão de condições agudas e crônicas^(37-39)^.

### Limitações do estudo

O estudo tem limitações relacionadas à coleta por conveniência e ao uso de gravações de consultas, que podem causar constrangimento aos participantes, para além do risco de viés do observador. A interpretação dos achados também é limitada pelo escopo da produção científica disponível, uma vez que a literatura brasileira pouco detalha a atuação prática do enfermeiro da ESF em promoção de saúde, e a literatura internacional não especifica o papel do EPA nesse contexto. Também houve limitações pela ausência de dados secundários sobre atendimentos a idosos, questões de gênero/raça, saúde de trabalhadores rurais e indígenas e outros aspectos importantes, que poderiam enriquecer a análise. Por fim, cabe destacar que o instrumento foi baseado no referencial teórico e no conhecimento dos pesquisadores.

### Contribuições para a área da enfermagem, saúde ou política pública

O presente estudo contribui para a fundamentação das competências necessárias ao enfermeiro da Atenção Primária à Saúde para o atendimento da população feminina, especialmente no que se refere às práticas destinadas à promoção da saúde e prevenção de doenças, alinhadas aos princípios das PAE. A análise e sistematização dessas competências nas consultas de enfermagem oferecem evidências relevantes que podem subsidiar a qualificação profissional e orientar a formulação de diretrizes e políticas públicas voltadas à futura implementação das PAE no contexto brasileiro. Os achados também favorecem o fortalecimento da prática avançada e impulsionam o desenvolvimento técnico-científico da enfermagem no país.

## CONSIDERAÇÕES FINAIS

Os resultados deste estudo evidenciam lacunas no desenvolvimento das competências relacionadas às PAE no domínio da promoção da saúde e prevenção de doenças durante as consultas de enfermagem voltadas à saúde das mulheres na APS. As fragilidades identificadas na atuação profissional evidenciam a necessidade de fortalecer essas competências desde a formação acadêmica, com foco na prática clínica e no cuidado centrado na pessoa.

Para que as competências das PAE em saúde da mulher possam ser plenamente incorporadas à prática clínica, é fundamental promover a reestruturação da formação e do exercício profissional, por meio de respaldo técnico-científico e reconhecimento legal dos órgãos competentes. Nesse sentido, a implementação das PAE no Brasil demanda investimentos em formação especializada, baseada em evidências e orientada pelas necessidades de saúde das mulheres, visando consolidar uma prática clínica ampliada e resolutiva na promoção da saúde e prevenção de doenças.

## Data Availability

Os dados de pesquisa só estão disponíveis mediante solicitação.
